# CO_2_ upgrading into bioproducts using a two-step abiotic–biotic system

**DOI:** 10.1073/pnas.2512565122

**Published:** 2025-08-18

**Authors:** Geonhui Lee, Hye-Jin Jo, Jihoon Choi, Maria Fonseca Guzman, Yu Shan, Han K. D. Le, Julian Feijoo, Nathan Soland, Douglas S. Clark, Peidong Yang

**Affiliations:** ^a^Department of Chemistry, University of California Berkeley, Berkeley, CA 94720; ^b^Department of Chemical and Biomolecular Engineering, University of California Berkeley, Berkeley, CA 94720; ^c^Department of Materials Science and Engineering, University of California Berkeley, Berkeley, CA 94720; ^d^Molecular Biophysics and Integrated Bioimaging Division, Lawrence Berkeley National Laboratory, Berkeley, CA 94720; ^e^Materials Sciences Division, Lawrence Berkeley National Laboratory, Berkeley, CA 94720

**Keywords:** CO_2_ fixation, abiotic-biotic, electrocatalysis

## Abstract

The valorization of CO_2_ to chemicals beyond C_1-2_ products is receiving significant interest; however, the direct electrosynthesis of C_n_ molecules (n > 4) remains a challenge. Here, we present a two-step abiotic–biotic system for upgrading CO_2_ into the biopolymer, poly(3-hydroxybutyrate). In the electrolysis system, CO_2_ is converted into C_2_ oxygenates using a Cu–Ag tandem electrocatalyst. The electrolysis process generates a liquid stream containing ~ 200 mM acetate in a bio-compatible electrolyte. This electrosynthesized acetate is then fed to a bioreactor, where the substrate is upgraded by *Cupriavidus necator* to biopolymer with a maximum rate of 32 ± 3.5 mg L^−1^ h^−1^. We further demonstrate the purification of the resulting biopolymer into a powder. The high productivity of the abiotic–biotic system demonstrates its feasibility for sustainable chemical manufacturing.

## Significance

The chemical industry faces a significant challenge with CO_2_ emissions due to its heavy reliance on fossil fuels. Electrochemical CO_2_ upgrading powered by renewable electricity presents a promising strategy to reduce hard-to-abate CO_2_ emissions. However, the direct electrosynthesis of chemicals beyond C_1-2_ products has been limited. Developing effective approaches for producing higher energy density molecules, such as fuels, fine chemicals, and biopolymers, is of significant importance. This work introduces a two-step abiotic–biotic system for upgrading CO_2_ into the biopolymer, poly(3-hydroxybutyrate). By doing so, the system leverages both the high reaction rates of electrochemical CO_2_ conversion to C_2_ oxygenates and the energy-efficient biological upgrading of these C_2_ intermediates into biopolymers.

**Fig. 1.**
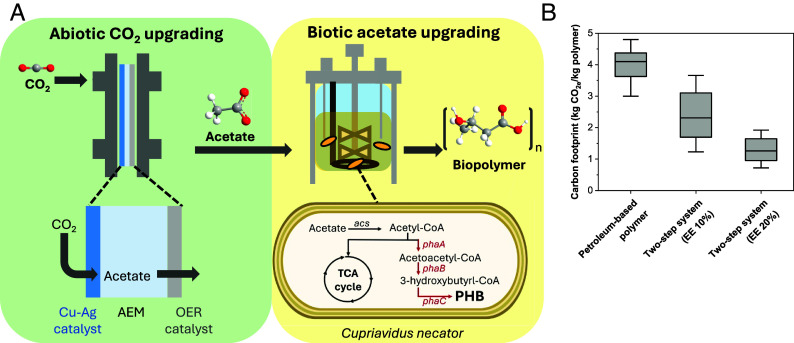
CO_2_ upgrading into bioproducts using the abiotic–biotic system. (*A*) Process flow for abiotic–biotic conversion of CO_2_ to chemicals and fuels. CO_2_ is electrochemically upgraded into C_2_ oxygenates (such as acetate and ethanol) at the cathode, which are then concentrated in a bio-compatible anolyte. These oxygenates are supplied to a bioreactor where bacteria convert these substrates into valuable bioproducts. The schematic of *Cupriavidus necator* (*C. nectar*) shows poly(3-hydroxybutyrate) (PHB) biosynthesis from acetate. Acetate is taken up by *C. necator* and converted to acetyl-CoA via acetyl-CoA synthetase (*acs*). Acetyl-CoA enters the TCA cycle for energy generation or serves as a precursor for PHB biosynthesis. In the PHB biosynthetic pathway, PhaA catalyzes the condensation of two molecules of acetyl-CoA to form acetoacetyl-CoA, which is then reduced to 3-hydroxybutyryl-CoA by PhaB. Finally, PhaC polymerizes 3-hydroxybutyryl-CoA into PHB. Red text indicates genes encoding enzymes involved in PHB biosynthesis (17). (*B*) Carbon footprint comparison between petroleum-based polymers and the two-step system. The two-step system involves the abiotic–biotic conversion of CO_2_ into PHB, with energy efficiency (EE) referring to the CO_2_ electrolyzer. In both cases, the yield of the bioreactor is assumed to be 50%.

The chemical industry faces a significant challenge with CO_2_ emissions due to its heavy reliance on fossil fuels. While many strategies for replacing fossil fuels with biomass feedstocks for biological fermentation have been proposed and are in various stages of development (1–3), they often compete with the human food chain and require extensive land use (4–6). Alternatively, an approach involving electrochemical upgrading powered by renewable electricity offers the potential to reduce hard-to-abate CO_2_ emissions. Yet, to date, direct electrosynthesis of long-chain molecules (C_4+_) from CO_2_ has only achieved a current density of <2 mA cm^−2^ (7–10), compared to C_2_ oxygenates (ethanol and acetate) that have reached industrially relevant current densities exceeding 100 mA cm^−2^ (11–13). Such high current densities motivated us to explore an approach combining an electrochemical system for CO_2_-to-C_2_ oxygenates conversion with a microbial system that utilizes C_2_ oxygenates to produce chemicals beyond C_1-2_ products (Fig. 1*A*). The electrosynthesized C_2_ oxygenates serve as viable substrates for microorganisms, allowing them to consume a liquid feedstock more efficiently than gaseous molecules and produce diverse value-added products (14–16).

The abiotic–biotic system couples electrochemical CO_2_ conversion with biological polymer synthesis, offering a promising route for sustainable biopolymer production. Herein, we offer an analysis that estimates the carbon footprint of the two-step process under projected performance conditions (Fig. 1*B* and *SI Appendix*, *Supplementary Note* 1). For large-scale commodity production, microbial productivity is projected to reach 2 to 4 g L^−1^ h^−1^ (16–20). Assuming a 50% yield in the bioreactor and a 20% energy efficiency of CO_2_ electrolyzer (18, 20), the two-step process contributes to 0.7 to 2 kg CO_2eq_/kg polymer depending on the renewable electricity sources used. The major source of carbon emission is the CO_2_ electrolyzer, as the bioreactor has relatively low heat and electricity demand. This carbon footprint is lower than that of petroleum-based polymers, which is ~4 kg CO_2eq_/kg polymer (21). This estimate suggests that abiotic–biotic upgrading has the potential for sustainable chemical manufacturing.

Several studies have explored CO_2_ conversion to bioproducts using a sequential process of CO_2_ electrolysis followed by microbial fermentation (22–27). Among CO_2_-derived products, acetate has been considered a key substrate since it forms acetyl-CoA that participates in various biological valorization processes (14, 28–30). The acetate stream is typically generated through a tandem process: electrolysis of CO_2_ to CO, followed by CO-to-acetate conversion, due to the unfavorable reaction pathway of CO_2_ to acetate (31–34). However, this approach adds complexity to the system and increases the costs associated with CO/CO_2_ separation (29, 35, 36). Furthermore, the downstream operation of the CO_2_ electrolyzer poses a challenge, as liquid products must be separated from a concentrated electrolyte (~10 M), which is detrimental to microorganisms due to excessive salts (37). Previous studies addressed this issue by introducing the adjustable effluent in an additional layer or using a diluted electrolyte (25–27). However, these strategies increase resistance within the systems, resulting in higher costs (29). Therefore, selecting a biocompatible electrolyte while maximizing the overall system efficiency is a key design principle for coupling CO_2_ electrolysis with microbial fermentation.

## Results

Here, we present a proof-of-concept demonstration of a two-step abiotic–biotic system that upgrades CO_2_ into PHB (Fig. 1*A*). First, CO_2_ electrolysis delivers sufficient acetate with a target condition of > 72 mA cm^−2^ (*SI Appendix*, *Supplementary Note* 2). To this end, we employed a Cu–Ag tandem electrocatalyst and a membrane electrode assembly (MEA) cell with an anion exchange membrane (AEM). In the AEM/MEA system, liquid products cross the AEM; acetate, a negatively charged ion, is transported to the anodic side. (*SI Appendix*, Fig. S1) The generated liquid stream from the anodic outlet, composed of electrosynthesized acetate and anolyte, was directly used as the electrolyte for biosynthesis. As detailed in *SI Appendix*, Table S5, this solution was supplemented only with minimal nutrients necessary for bacterial growth, while electrosynthesized acetate served as the sole carbon source. In the subsequent bioupgrading stage, *C. necator* is employed to utilize the acetate in the electrosynthesized solution as a carbon source for PHB production. In this process, *C. necator* converts acetate to acetyl-CoA by acetyl-CoA synthetase, and the resulting acetyl-CoA enters *C. necator*’s native metabolic pathway involving three enzymes encoded in one operon, PhaA, PhaB, and PhaC, to synthesize PHB (17). The abiotic–biotic system, which divides the process into two separate steps (converting CO_2_ to acetate, then converting acetate to PHB), circumvents challenges such as media conditions unsuitable for bacterial growth, thus allowing each step to be optimized independently.

In prior studies, we demonstrated that a tandem Cu–Ag electrocatalyst enhances the production rate of C_2_ oxygenates (38, 39). Ag supplies CO intermediates to catalytic active sites, facilitating C–C coupling and shifting selectivity toward oxygenate products. Yet, these systems have been limited by low selectivity and reaction rates below the target current density of > 72 mA cm^−2^. The MEA system, equipped with a gas diffusion electrode (GDE), directly supplies gas-phase CO_2_ to the electrocatalysts, allowing CO_2_ electrolysis to operate at a higher current density, approaching sub-A cm^−2^. Furthermore, reactant concentration can be tuned by exploiting mass transport control (40–42).

**Fig. 2.**
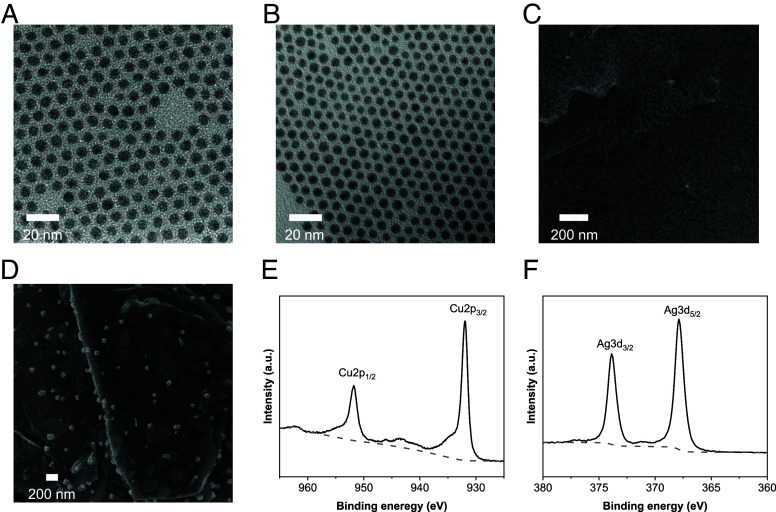
Morphology and characterization of the Cu–Ag tandem electrocatalyst. (*A* and *B*) Transmission electron microscopy images of (*A*) 7 nm Cu nanoparticles and (*B*) 6 nm Ag nanoparticles. (*C* and *D*) Scanning electron microscope images of (*C*) as-prepared Cu–Ag tandem electrocatalyst on carbon paper-based gas diffusion electrode and (*D*) post-electrolysis of Cu–Ag tandem electrocatalyst on carbon paper-based gas diffusion electrode. (*E* and *F*) X-ray photoelectron spectroscopy spectra of (*E*) Cu of the as-prepared Cu–Ag tandem electrocatalyst and (*F*) Ag of the as-prepared Cu–Ag tandem catalyst.

We proceeded by implementing these concepts in combination. We prepared nanoscale copper and silver nanoparticles (NPs) using our group’s prior metho (43, 44) and employed the Cu–Ag bimetallic NPs on the carbon paper-based GDE. The 7 nm Cu NPs and 6 nm Ag NPs were synthesized via colloidal synthesis, with a narrow size distribution, as shown in transmission electron microscopy (TEM) images (Fig. 2 *A* and *B*). The as-synthesized Cu and Ag NPs were mixed in hexane and coated on the GDE using an air-brush method. GDE was prepared by airbrushing a mixture of 20-μm-sized graphite and Nafion binder onto a commercial carbon paper. Scanning electron microscopy (SEM) images show the islands of Cu–Ag NPs assembled on the graphite flakes (Fig. 2*C* and *SI Appendix*, Fig. S2 *A* and *B*), and these Cu–Ag NPs agglomerate into larger particles than the original size (6 to 7 nm), forming a close-contact network during electrolysis (Fig. 2*D* and *SI Appendix*, Fig. S2 *C* and *D*). In previous work, our group found that Cu and Ag NPs migrate and agglomerate, as revealed by *operando* and postelectrolysis microscopic studies (39, 44). In the MEA system, we also observed the reconstruction of Cu–Ag NPs. Energy-dispersive X-ray spectroscopy (EDX) with a transmission electron microscope (TEM) for post-electrolysis electrodes shows that Ag nanodomains become embedded within the Cu domains (*SI Appendix*, Fig. S3). In the network, Ag serves as a CO-producing electrocatalyst, while C–C coupling occurs at metallic nanodomains, producing a mixture of C_2+_ products (acetate, ethylene, ethanol, and propanol). To investigate how the CO generation/consumption rate affects the selectivity in the CO_2_ reduction reaction (CO_2_RR) systems, we prepared various ratios of Ag at 0, 20, 45, and 66%. The surface composition of Cu–Ag was confirmed by inductively coupled plasma optical emission spectrometry (ICP-OES) (*Materials and Methods* and *SI Appendix*, Table S3).

**Fig. 3.**
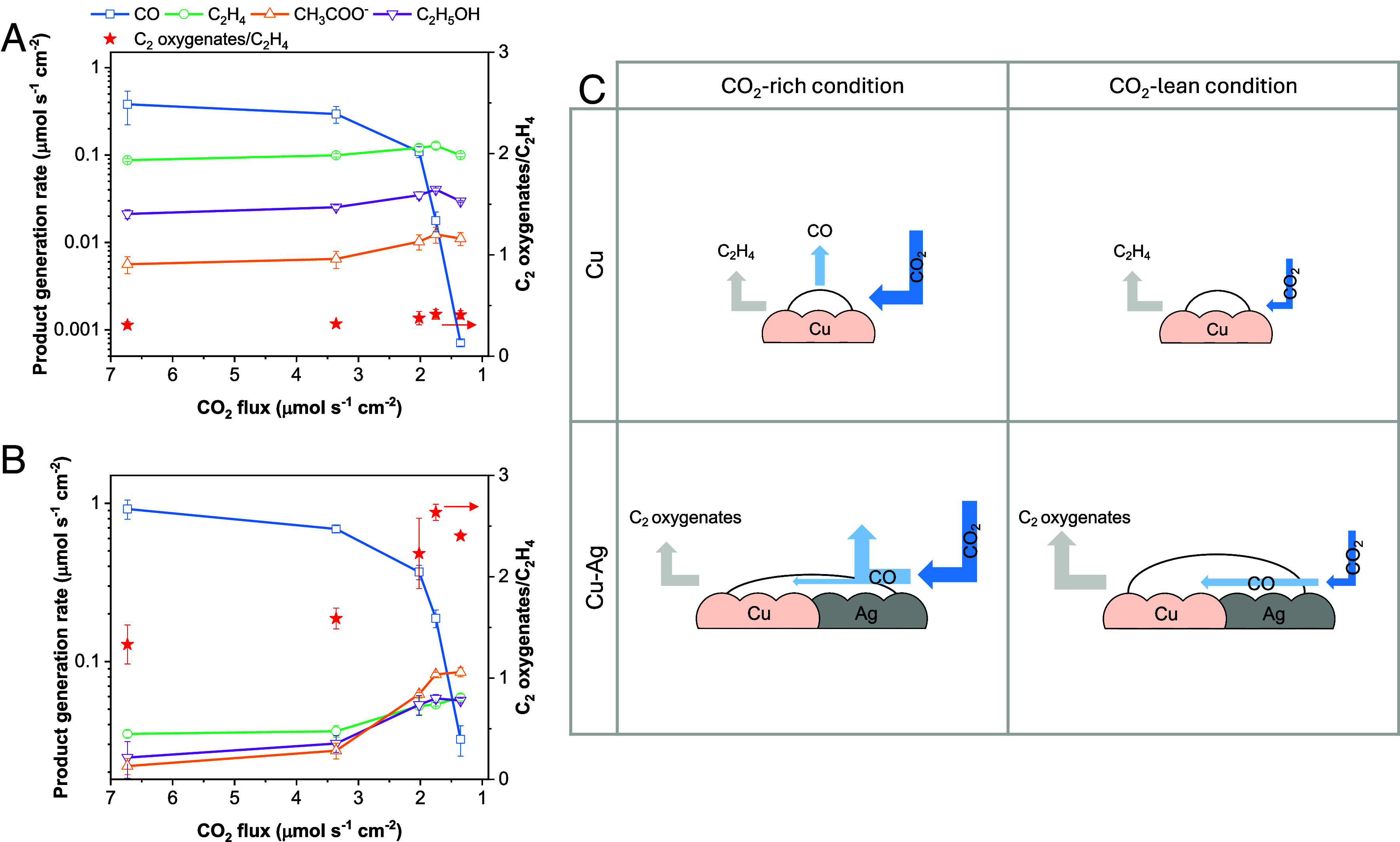
CO coverage-dependent selectivity of C_2_ products. (*A* and *B*) Product generation rate and the ratio between C_2_ oxygenates and C_2_H_4_ for different CO_2_ fluxes at applied current density 300 mA cm^−2^ in the MEA cell (*A*) with pure Cu electrocatalyst (*B*) with Cu–Ag (Ag 45%) tandem electrocatalyst. (*C*) Schematic of species flow and CO coverage on the electrocatalyst surface under CO_2_-rich and CO_2_-lean conditions. A CO_2_-rich condition corresponds to high CO_2_ flux, while a CO_2_-lean condition corresponds to low CO_2_ flux.

We conducted CO_2_ electrolysis using Cu–Ag tandem electrocatalysts in the MEA system. The GDE with Cu–Ag tandem electrocatalysts, the AEM, and IrO_x_/Ti felt were pressurized between two metal plates. High-purity CO_2_ gas was fed to the cathode, and 0.1 M CsH_2_PO_4_ was used as the anolyte. We then applied constant current in the range of 100 to 400 mA cm^−2^ to the MEA cell. However, CO was the dominant product while C_2_ oxygenates FE remained < 20% when using Cu–Ag electrocatalysts at a CO_2_ flow rate of > 3.4 μmol s^−1^ cm^−2^ (5 sccm cm^−2^) (*SI Appendix*, Fig. S7). We posited that the limited residence time of CO at the electrocatalyst surface, due to the high CO_2_ flux, prevented CO from being consumed for C–C coupling (40, 41). We tested this hypothesis by decreasing the CO_2_ flow rate until all CO_2_ could be consumed for C_2_ product formation; for example, the minimum CO_2_ flux for 8e^-^ products and 12e^-^ products is 2.1 μmol s^−1^ cm^−2^ at 300 mA cm^−2^ with a theoretical CO_2_ conversion efficiency in the alkaline CO_2_ electrolysis system (*SI Appendix*, *Supplementary Note* 3) (45). In the CO_2_ flux range of 1 to 7 μmol s^−1^ cm^−2^, we observed an increase in C_2_ FE as CO_2_ flux decreased for both Cu and Cu–Ag systems (Fig. 3 *A* and *B*). As CO_2_ flux decreases, CO molecules remain longer at C–C coupling active sites, enhancing C_2_ conversion efficiency. However, H_2_ FE increases at the point where CO_2_ is fully depleted (*SI Appendix*, Fig. S8). Product distribution was varied with different electrocatalysts. For Cu, C_2_H_4_ is the major product, with FE of 34 to 49%, while C_2_ oxygenates production overwhelms C_2_H_4_ by using Cu–Ag (Ag 32%). The ratio of C_2_ oxygenates and C_2_H_4_ (the carbon selectivity of C_2_ oxygenates/C_2_H_4_) is higher for Cu–Ag than for Cu at all CO_2_ inputs. Among C_2_ oxygenates, a substantial increase in CH_3_COO^−^ production was observed, followed by C_2_H_5_OH, as the CO_2_ flux decreased from 6.7 μmol s^−1^ cm^−2^ to 1.8 μmol s^−1^ cm^−2^. The highest FE for C_2_ oxygenates was achieved with CH_3_COO^-^ FE of 21% and C_2_H_5_OH FE of 23% at the CO_2_ flux of 1.8 μmol s^−1^ cm^−2^. The shift in selectivity from C_2_H_4_ to CH_3_COO^−^ in the tandem system is driven by the increased rate of CO generation rate from Ag, which creates CO_2_-lean/CO-enriched microenvironments and boosts *CO coverage on the Cu–Ag surface (Fig. 3*C*) (42, 46, 47).

**Fig. 4.**
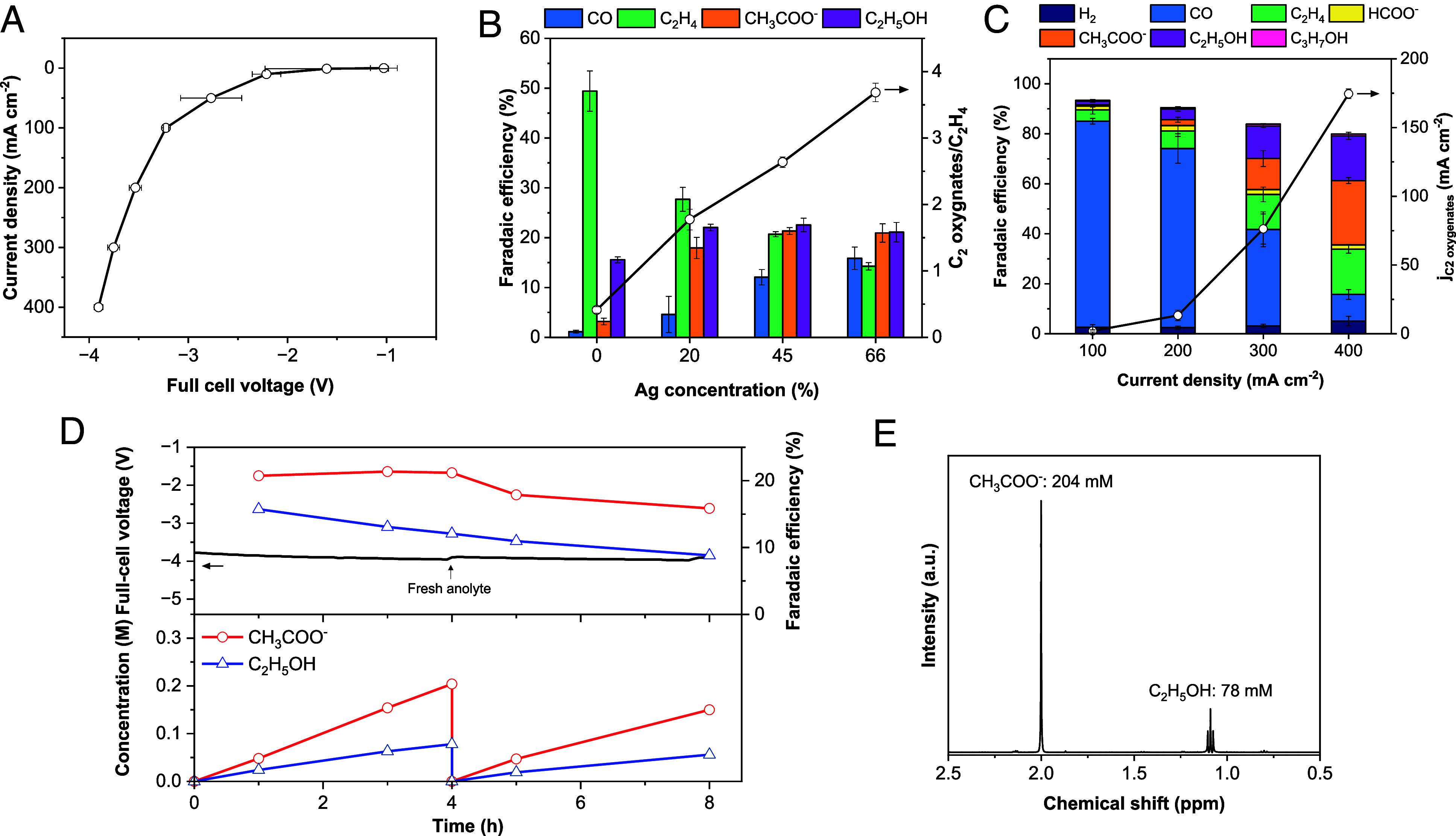
Electrochemical performance of Cu–Ag tandem electrocatalyst and electrosynthesized C_2_ oxygenates stream. (*A*) Full-cell jV of Cu–Ag tandem electrocatalyst in the MEA cell. (*B*) Product distribution and the ratio between C_2_ oxygenates and C_2_H_4_ of Cu–Ag tandem electrocatalysts with varying Ag ratios (0, 20, 45, and 66%) at applied current density 300 mA cm^−2^. (*C*) Product distribution of Cu–Ag (Ag 45%) tandem electrocatalyst at applied current density from 100 mA cm^−2^ to 400 mA cm^−2^ and the partial current density of acetate and ethanol. (*D*) Long-term operation for Cu–Ag tandem electrocatalyst and concentration of collected acetate and ethanol. (*E*) NMR spectra of the collected C_2_ oxygenate stream.

We further studied Cu–Ag ratios to gain insights into how *CO coverage tunes the reaction pathway of C_2_ products. Pure Cu and Cu–Ag electrocatalysts with Ag contents of 20%, 45%, and 66% were tested at 1.8 μmol s^−1^ cm^−2^ and an applied current density of 300 mA cm^−2^. As shown in Fig. 4*B*, increasing Ag content at the catalytic sites boosts the CO generation, which in turn raises the C_2_ oxygenates/C_2_H_4_ from 0.4 to 3.7. At higher Ag concentration of 66%, total C_2_ FE drops due to a lack of C–C coupling sites; however, the C_2_ oxygenates/C_2_H_4_ is 3.7, compared to 0.4 for pure Cu. These results suggest that *CO coverage, enhanced by CO-producing catalysts, promotes C–C coupling, particularly towards CH_3_COO^−^ rather than C_2_H_4_. As a result, using the Cu–Ag (Ag 45%) tandem electrocatalyst under limited CO_2_ flux conditions, we achieved 44% C_2_ oxygenates FE, with 26% for CH_3_COO^−^ and 18% for C_2_H_5_OH, at 400 mA cm^−2^ and −3.9 V_full-cell_ (Fig. 4 *A* and *C*). The partial current density for C_2_ oxygenate products reaches 176 mA cm^−2^, exceeding the target production rate of 72 mA cm^−2^ for microbial biotic upgrading on an industrial scale.

We sought to collect C_2_ oxygenate products in the bio-compatible anolyte (0.1 M KH_2_PO_4_) until their concentration reached a level that remains within the acetate tolerance range for microorganisms (48–50). We collected 200 mM of acetate and 80 mM of ethanol in 30 mL of anolyte over 4 h at the applied current density of 300 mA cm^−2^, with the C_2_ oxygenates FE in the range of 35 to 39%. (Fig. 4*D*) The C_2_ oxygenates FE decreases by 5% due to the cation change from Cs^+^ to K^+^ (*SI Appendix*, Fig. S13) (51, 52). However, since Cs^+^ inhibits bacterial growth by interfering with K^+^ uptake and disrupting intracellular ion homeostasis, we used KH_2_PO_4_ as the anolyte (*SI Appendix*, Fig. S13) (53). The concentration of C_2_ oxygenates steadily increases, with no significant loss of CH_3_COO^−^ or C_2_H_5_OH due to oxidation. The pH of the anolyte decreased from 4.6 to 2.7, but the full-cell voltage remained stable, with only a deviation of ~0.3 V. To ensure microbial compatibility, the anolyte was neutralized to pH 7.0 before inoculation. We circulated the anolyte at a volume of 5 to 10 mL cm^−2^, depending on the operation time and replaced it with fresh solution whenever the concentration of CH_3_COO^−^ reached the desired level of > 200 mM, which is the minimum concentration for the sequential biotic upgrading (Fig. 4*E*). To feed sufficient electrosynthesized liquid products, we extended the operation time. As a result, we collected liquid products reaching ~200 mM of acetate and ~100 mM of ethanol in ~470 mL over 129 h (*SI Appendix*, Table S4), and the mixtures of liquid products and electrolyte were fed to the microorganism after adding the optimal medium and adjusting pH (*Materials and Methods*). In the solution, inorganic species, such as Cu, Ag, and Ir, were detected at concentrations below ~100 ppb (*SI Appendix*, Table S2).

**Fig. 5.**
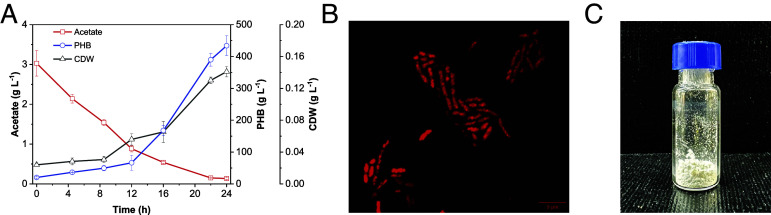
Bioconversion of electrosynthesized acetate to PHB using *C. necator.* (*A*) Time-course profiles of acetate consumption (red, *Left y*-axis), PHB production (blue, *Right y*-axis), and Biomass (cell dry weight (CDW), black, *Right y*-axis) during the bioconversion process. Data represent mean values ± SD from triplicate experiments. (*B*) Confocal fluorescence microscopy image of *C. necator* stained with Nile red, showing intracellular PHB accumulation as bright fluorescence signals. (Scale bar, 5 μm.) The sample was collected at 24 h and concentrated to OD 30 before imaging. (*C*) Purified PHB powder was obtained after extraction using 1,3-dioxolane, yielding 61 mg of a white powder.

As a proof-of-concept for a two-step system, we sought to feed electrosynthesized acetate to *C. necator* to produce PHB on a laboratory scale. Electrosynthesized acetate served as the carbon source for both *C. necator* growth and PHB accumulation. Based on previous studies, an acetate concentration of 3 g L^−1^ was selected as optimal. (48) In addition, the medium was supplemented with other essential components, and the pH was adjusted to 7.0 prior to inoculation. This bioconversion process transforms acetate into PHB via its conversion to acetyl-CoA, followed by polymerization through its well-known native pathway. Both the cell dry weight (CDW) and PHB accumulation in *C. necator* increased over time, with a notable increase observed after the initial lag phase. Despite a slightly longer lag phase than that observed with standard sodium acetate (*SI Appendix*, Fig. S14), the overall PHB production yield remained comparable, further supporting the suitability of the electrosynthesized acetate for microbial biopolymer biosynthesis. The results of the bioconversion process demonstrate PHB production (Fig. 5*A*) by 24 h, when the PHB concentration reached 434 ± 31 mg L^−1^ and the cell concentration increased to 0.14 ± 0.0065 g L^−1^. The corresponding PHB content of the cells was 75 ± 1.6 weight %. The highest PHB production rate was observed between 12 and 22 h, reaching 32 ± 3.5 mg L^−1^ h^−1^. To further investigate intracellular PHB accumulation, confocal fluorescence microscopy was performed after staining the cells with Nile red, a lipophilic dye that selectively binds to PHB granules. (54) The confocal images clearly show distinct fluorescence signals within the cells, qualitatively confirming PHB formation (Fig. 5*B*). This visual evidence supports the quantitative measurements, demonstrating that the electrosynthesized acetate solution effectively sustains microbial PHB biosynthesis. Furthermore, to obtain PHB in purified form, the polymer was extracted from harvested cells using 1,3-dioxolane, a green solvent known for its efficient PHB dissolution. (55) The extraction yielded 61 mg of PHB as a fine powder, further demonstrating its successful biosynthesis and accumulation. These findings highlight the potential of the two-step system for converting CO_2_ into value-added biopolymers.

## Discussion

The combination of catalyst design and microenvironment control enables us to electrosynthesize C_2_ oxygenates at industrially relevant production rates. The electrolysis system continuously generates a C_2_ oxygenates stream in the bio-compatible electrolyte, which is subsequently fed to *C. necator* in the bioreactor. This approach successfully demonstrates the production of fine biopolymer powder from electrosynthesized acetate.

The carbon footprint of the lab-based two-step process is 1.5 to 4.6 kg CO_2e_/kg polymer, depending on the source of renewable electricity (*SI Appendix*, Table S1). Thus, we note the need for further advances in electrocatalyst design to enhance the selectivity of acetate and reduce the overpotential to improve energy efficiency.

On the biological side, metabolic and reaction engineering efforts are required to increase the productivity of acetate-to-PHB conversion, and system architecture design is key for efficiently integrating abiotic and biotic components. In addition, expanding the platform to incorporate other microbial hosts (56) or engineered pathways (57–59) capable of utilizing both acetate and co-produced carbon compounds (e.g., ethanol) could further increase carbon conversion efficiency.

## Materials and Methods

### Catalyst Preparation and Characterization.

All reagents used in this work were purchased from suppliers without further purification. Then, 7 nm Cu and 6 nm Ag nanoparticles were synthesized using our group’s previous method (39, 44). Both nanoparticles were dispersed in hexane. The concentration of Cu and Ag in hexane was analyzed by inductively coupled plasma optical emission spectroscopy (PerkinElmer Optima 7000 DV). The mixed solutions with different Cu–Ag ratios were prepared considering the concentration of Cu and Ag. The atomic composition of the as-prepared GDEs was measured by ICP-OES (*SI Appendix*, Table S3). The ICP samples are prepared by redispersing the NPs from the GDEs in 20% HNO_3_. The sizes and shapes of the as-prepared NPs were confirmed by transmission electron microscopy (Hitachi H-7650) and SEM (Ultra 55-FESEM). For TEM-EDX measurements (at 300 kV on the FEI ThemIS microscope), NPs were redispersed in ethanol by ultrasonication, then deposited on the TEM grids.

### Electrode Preparation.

The cathode was prepared by airbrushing graphite ink and Cu–Ag ink onto carbon paper (GDS5130, AvCarb). Graphite ink was prepared with 1 mg of 20 μm graphite (Sigma-Aldrich) and 10 μL of Nafion binder (D520CS, Ionpower) in Isopropyl alcohol (IPA) and then was sonicated for 1 h. The mass loading of graphite is 0.05 mg cm^−2^. The pure Cu NPs or Cu–Ag NPs were dispersed in hexane, and the solution was sprayed onto the graphite/carbon paper until the mass loading reached 0.05 mg cm^−2^. The cathode was dried for 24 h in a vacuum chamber.

### Electrochemical Experiments.

The electrochemical data were collected using an electrochemical station (biologics). The 5 cm^2^ MEA cell (dioxide materials) was used. The cathode and anode were separated by an AEM (Sustainion® X37-50 grade RT Membrane). The anode, an oxygen evolution reaction catalyst, is titanium felt-supported iridium oxide (Magneto). The anolyte is 0.1 M CsH_2_PO_4_ or KH_2_PO_4,_ and it was circulated using a peristaltic pump. CsH_2_PO_4_ was prepared by mixing Cs_2_CO_3_ or CsHCO_3_ and H_3_PO_4_. Humidified CO_2_ gas is fed with a mass flow from 2 to 10 sccm cm^−2^. Electrolysis was maintained for at least 30 min before collecting gas and liquid samples. The experiments were repeated at least 3 times for the average and SE.

The gas-phase products were analyzed by gas chromatography (Agilent Technologies, 7890B) equipped with a thermal conductivity detector (TCD) and a flame ionization detector (FID). The liquid-phase products were analyzed with a 500 MHz Bruker Avance IV NEO. We prepared the liquid sample with 100 μL of dimethyl sulfoxide (DMSO) standard solution (300 μM ~ 16 mM depending on the liquid product concentrations), 100 μL of post-electrolysis electrolyte, and 400 μL of D_2_O. We conducted proton NMR (HNMR) and compared the areas of proton peaks of DMSO (~ 2.6 ppm), formate (~ 8.1 ppm), ethanol (~ 1.1 ppm), acetate (~ 2 ppm), propanol (0.8 ppm) (*SI Appendix*, Fig. S11).

### Liquid Product Collections.

Long-term operation was conducted to collect sufficient liquid products. The cell configuration was the same as in electrochemical experiments. Liquid products are accumulated in 0.1 M KH_2_PO_4_ anolyte over 4 ~ 15 h. The volume of anolyte (20~50 mL) was adjusted by operation time, and replaced with fresh solution whenever the concentration of CH_3_COO^−^ reached the desired level of ~ 200 mM. The cathode was changed when the performance degradation was observed. The final concentration and volume of 15 cycles—a cycle with fresh electrolyte—are 0.209 mM and 468 mL, respectively.

### Microbial Experiments.

*C. necator* (ATCC 17699) was obtained from the American Type Culture Collection (ATCC). Prior to experimentation, a single colony was inoculated into 5 mL of Luria-Bertani (LB) broth and cultivated overnight at 30 °C, 200 rpm. The overnight culture was used to inoculate 50 mL of M9 minimal medium containing fructose as the carbon source in a 250-mL baffled Erlenmeyer flask. The culture was incubated at 30 °C, 200 rpm for 12 to 16 h until the exponential growth phase was reached. Cells were harvested by centrifugation at 4,000×*g* for 10 min at 4 °C, washed twice with sterile phosphate-buffered saline (PBS, pH 7.0), and resuspended in fresh DM9 containing electrochemically synthesized acetate (3 g L^−1^) as the sole carbon source (*SI Appendix*, Table S5).(60) The initial optical density at 600 nm (OD_600_) was adjusted to 0.5. Nitrogen limitation was applied to promote PHB accumulation by supplementing ammonium chloride (NH_4_Cl) at a concentration of 0.09 g L^−1^, maintaining a carbon-to-nitrogen (C/N) molar ratio of approximately 30. (61) All cultivations were performed in triplicate. Bacterial growth and PHB production were monitored at designated time points by measuring OD_600_ and PHB concentrations.

### Product Analysis Using High-Performance Liquid Chromatography (HPLC).

Cell pellets were collected from 5 mL of culture by centrifugation at 4,000 × *g* for 10 min. The harvested pellets were lysed, and the PHB was depolymerized by adding 1 mL of concentrated sulfuric acid (H_2_SO_4_) and incubating at 95 °C for 60 min. The depolymerized PHB solution was then diluted with 4 mL of HPLC-grade water, and following the addition of adipic acid solution as an internal standard filtered through a 0.2 μm filter prior to analysis. PHB was quantified using a HPLC system (HPLC 1260 Infinity II, Agilent, CA) equipped with an Aminex HPX-87H column (300 × 7.8 mm; Bio-Rad). The analysis was conducted under the following conditions: sample injection volume, 10 μL; UV detection, 210 nm; flow rate, 0.6 mL/min; column temperature, 40 °C.

### Confocal Microscopy.

Intracellular PHB granules in *C. necator* were visualized using confocal laser scanning microscopy. (62) Bacterial cultures were collected by centrifugation, washed twice with sterile phosphate-buffered saline (PBS, pH 7.0), and resuspended in PBS to an OD_600_ of 30. For fluorescence staining, 3 μL bacterial suspension was mixed with 0.3 μL Nile Red solution (1 mg/mL in dimethylsulfoxide) and incubated for 1 h at room temperature. Twenty μL of a 2% (w/v) low-melting agarose solution (preheated to 60 °C) was applied for fixation, followed by 1 μL of the stained cell suspension. Fluorescence imaging was performed using a Zeiss LSM880 confocal laser scanning microscope (Zeiss, Baden-Württemberg, Germany). PHB granules stained with Nile Red were visualized using an excitation wavelength of 514 nm, with fluorescence emission recorded in the range of 540 to 750 nm.

### PHB Extraction.

PHB extraction was performed following the protocol described by Wongmoon and Napathorn with slight modifications.(55) PHB extraction was performed using a shaking water bath method with 1,3-dioxolane as the extraction solvent. A 5% (w/v) wet cell suspension was prepared in 2 mL of 1,3-dioxolane and transferred to a toed glass tube sealed with a butyl rubber stopper and aluminum crimp. The extraction was conducted at 80 °C for 6 h in a shaking water bath at 100 rpm to ensure efficient polymer dissolution. Following extraction, PHB was recovered by adding three volumes of water to the solution, which induced phase separation. The PHB-enriched phase was collected by centrifugation at 4,000×*g* for 5 min at room temperature, and the harvested PHB was washed twice with water to remove residual solvent. The purified PHB was subsequently dried in an oven at 60 °C for 3 d to obtain the final powder.

## Supplementary Material

Appendix 01 (PDF)

## Data Availability

All study data are included in the article and/or *SI Appendix*.
