# Supplementary material for: CO2 upgrading into bioproducts using a two-step abiotic–biotic system
Source: Proc Natl Acad Sci U S A. 2025 Aug 18;122(34):e2512565122. doi: 10.1073/pnas.2512565122 (PMC12403087; doi:10.1073/pnas.2512565122)
Supplement: Supplementary file 1 — Appendix 01 (PDF) [file pnas.2512565122.sapp.pdf]

PNAS

Supporting Information for

CO_2 Upgrading into Bioproducts Using a Two-Step Abiotic-Biotic System

Geonhui Lee[1,2]†, Hye-Jin Jo[3]†, Jihoon Choi[5], Maria Fonseca Guzman[1], Yu Shan[5], Han Le[1], Julian Feijoo[1], Nathan Soland[1], Douglas S. Clark[3,4*], Peidong Yang[1,5,6*]

[1]Department of Chemistry, University of California Berkeley; Berkeley, CA 94720, USA

[2]Current address: School of Chemical and Biological Engineering, Seoul National University, Seoul, Republic of Korea

[3]Department of Chemical and Biomolecular Engineering, University of California Berkeley; Berkeley, CA 94720, USA

[4]Molecular Biophysics and Integrated Bioimaging Division, Lawrence Berkeley National Laboratory, Berkeley, CA, 94720, USA.

[5]Department of Materials Science and Engineering, University of California Berkeley; Berkeley, CA 94720, USA

[6]Materials Sciences Division, Lawrence Berkeley National Laboratory; Berkeley, CA 94720, USA

†These authors contributed equally to this work

*Corresponding authors. Douglas S. Clark, Peidong Yang
Email: p_yang@berkeley.edu; dsc@berkeley.edu

This PDF file includes:

Supporting text
Figures S1 to S14
Tables S1 to S5
SI References

Supporting Information Text

Supplementary Note 1. Carbon footprint analysis. We assessed the carbon footprint of the two-step system for biopolymer production under different energy efficiencies of the CO_2 electrolyzer and renewable electricity sources. The majority of energy consumption arises from CO_2 electrolysis, while the bioreactor has minimal heat and power demands – consuming only ~3% of the electricity required for electrolysis.(1) However, CO_2 is emitted at 0.25 g CO_{2e}/g polymer during microbial fermentation (eq. S3), thus, we add this CO_2 emission to the total carbon footprint.

The electrolysis energy cost is calculated using the following equation:

$$Electrolysis\ energy\ (J\ g^{-1}\ acetate) = \frac{8\ moles\ of\ electron}{1\ mole\ of\ acetate} \times 96485\ C\ mol^{-1} \times \frac{1}{59\ g\ mol^{-1}\ acetate} \times V_{full-cell} \times \frac{1}{Selectivity\ (\%)} \quad \text{(eq. S1)}$$

The carbon footprint is calculated using the following equation:

$$Carbon\ footprint\ (g\ CO_{2e}\ g^{-1}\ Polymer) = \left(\frac{Electrolysis\ energy + 0.03 \times Electrolysis\ energy\ (J\ g^{-1}\ acetate)}{Yield\ \left(\frac{g}{g}\right)}\right) \times 2.8E\text{-}7\ kWh\ J^{-1} \times carbon\ intensity\ of\ renewable\ electricity\ (g\ CO_{2e}\ kWh^{-1}) + 0.25\ g\ CO_{2e}\ g^{-1}\ Polymer \quad \text{(eq. S2)}$$

We present three scenarios in Table S1: 1) 10% energy efficiency at 3.5 $V_{full-cell}$, 400 mA cm^{-2} and 30% Faradaic efficiency for C_2 oxygenates, 2) 20% energy efficiency at 3.2 $V_{full-cell}$, 400 mA cm^{-2} and 56% Faradaic efficiency for C_2 oxygenates and 3) lab data with different renewable electricity sources including wind, hydro and solar electricity.(2, 3) The yield is considered at 50% in Table S1. The results illustrate how the performance of CO_2 electrolyzer affects the overall carbon footprint of the produced biopolymer.

Supplementary Note 2. Target current density. Here, we estimate the production rate of CO_2-derived products required to supply a suitable amount of feedstock to a bioreactor. In our study, we sought to produce polyhydroxybutyrate (PHB) by converting CO_2 into acetate using a membrane electrode assembly (MEA) system and subsequently upgrading acetate to the biopolymer with *Cupriavidus necator*.
We first calculate the theoretical carbon yield for acetate-to-PHB conversion according to the following chemical equation.(4)

$$9nCH_3COOH \rightarrow 4(C_4H_6O_2)_n + 6nH_2O + 2nCO_2 \quad \text{(eq. S3)}$$

The input carbon is

$$Input\ carbon = \frac{9n}{4}\ mol\ of\ acetate \times \frac{2\ moles\ of\ carbon}{mole\ of\ acetate} \times 12\frac{g}{mol\ carbon} = 54n\ g\ of\ carbon \quad \text{(eq. S4)}$$

The output carbon is

$$output\ carbon = n\ mol\ of\ PHB \times \frac{4\ moles\ of\ carbon}{mole\ of\ PHB} \times 12\frac{g}{mol\ carbon} = 48n\ g\ of\ carbon \quad \text{(eq. S5)}$$

We can calculate the theoretical yield of PHB carbon by dividing the output carbon by the input carbon:

$$Theoretical\ carbon\ yield = \frac{48\ n\ g\ of\ carbon\ in\ PHB}{54\ n\ g\ of\ carbon\ in\ acetate} = 88\% \quad \text{(eq. S6)}$$

The practical production of commodity chemicals generally targets 80% of the theoretical carbon yield.(3) In this study, we assume a 70% carbon yield in the bioreactor, which corresponds to a yield (g of PHB / g of acetate) of 50%. Furthermore, the microbial productivity required for commercialization is typically 2-4 g L^{-1} h^{-1}.(1, 5, 6)
We estimate the required production rate of acetate to meet these targets using the following equation:

$$Production\ rate\ of\ acetate\ (M\ h^{-1}) = \frac{PHB\ productivity\ (g\ L^{-1}\ h^{-1})}{Yield\ (g\ PHB/g\ acetate) \times 59.044\frac{g}{mol\ acetate}} \quad \text{(eq. S7)}$$

The necessary acetate production is 67-135 mM h^{-1}, as calculated in eq. S5. In our system, the acetate stream is delivered as a mixture of acetate and anolyte. We adjust the anolyte volume to 5 mL per cm^2 active area, which yields a target current density (mA cm^{-2}) in a range that maintains both an acceptable full-cell potential and high selectivity (~0.2 M acetate collection).
We use the following equation to calculate the current density for converting CO_2 to acetate:

$$Reaction\ rate\ (A\ cm^{-2}) = Production\ rate\ of\ acetate\ (mol\ L^{-1}\ h^{-1}) \times Anolyte\ volume\ (L\ cm^{-2}) \times \frac{8\ moles\ of\ electron}{1\ mole\ of\ acetate} \times 96485\ C\ mol^{-1} \times \frac{h}{3600\ s} \quad \text{(eq. S8)}$$

with assuming the anolyte volume of 5 mL cm^{-2} active area where the electrochemical reaction takes place.

The calculated reaction rate is 72-145 mA cm^{-2} for the microbial production rate of 2-4 g L^{-1} h^{-1} with the 50% yield. In practice, lab-based bioreactors often operate at ~30% yield, and the minimal anolyte volume may affect both full-cell potential and selectivity over extended operation. Therefore, further improvements to the production rate are required.

Supplementary Note 3. Theoretical CO_2 consumption

Assuming complete conversion of CO_2 into either acetate or ethanol, the minimum CO_2 flux is calculated using the following equation.

$$CO_2\ flux\ (mol\ s^{-1}\ cm^{-2}) = \frac{Current\ density\ (A\ cm^{-2})}{96485\ C\ mol^{-1}} \times \frac{1\ mole\ of\ product}{n\ moles\ of\ electrons} \times \frac{m\ moles\ of\ CO_2}{1\ mole\ of\ product} \times \frac{1}{Theoretical\ CO_2\ conversion\ efficiency\ (\%)} \quad \text{(eq. S9)}$$

Here, n is the number of electrons required for the CO_2 reduction reaction (CO_2RR): 8 moles of electrons are required to convert 2 moles of CO_2 into acetate, and 12 moles of electrons are required for CO_2-to-ethanol conversion. m represents the number of CO_2 moles that are involved in CO_2RR: 2 moles of CO_2 are required for both CO_2-to-acetae and CO_2-to-ethanol. A theoretical CO_2 conversion efficiency is 36% for CO_2-to-acetate and 25% for CO_2-to-ethanol in the alkaline CO_2 electrolysis system.(7)

The minimum CO_2 flux for $8e^-$ products and $12e^-$ products is 2.1 $\mu mol\ s^{-1}\ cm^{-2}$ and 2.1 $\mu mol\ s^{-1}\ cm^{-2}$ at 300 mA cm^{-2}, respectively. At 400 mA cm^{-2}, CO_2 flux is 2.9 $\mu mol\ s^{-1}\ cm^{-2}$ and 2.8 $\mu mol\ s^{-1}\ cm^{-2}$. In experiments, < 1% of CO_2 is detected at the cathodic outlet at < 2.1 $\mu mol\ s^{-1}$ and applied current density of 300 mA cm^{-2} (Fig. S6).

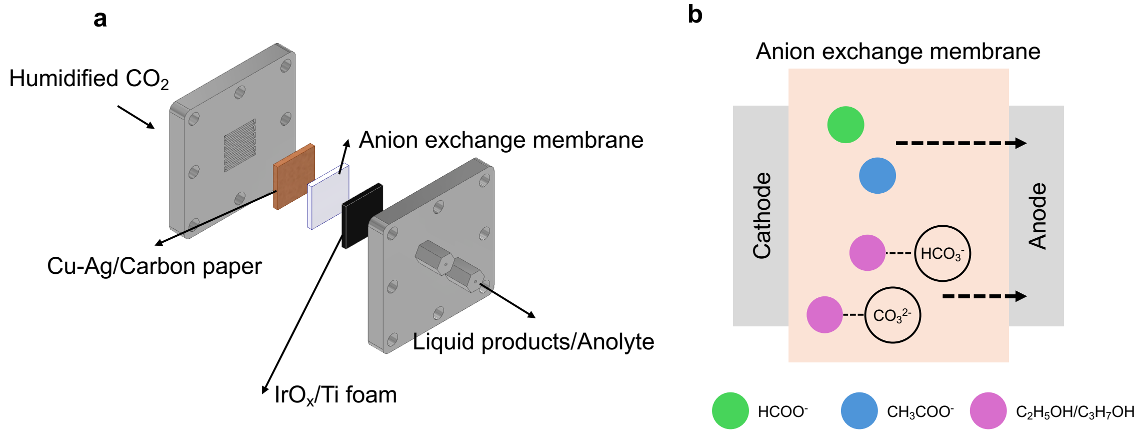

Fig. S1. a) Schematic of a membrane electrode assembly (MEA) cell. The cell components are metal plates, an anion exchange membrane (AEM), a cathode and an anode. b) Liquid product crossover in the anion exchange membrane (AEM)/MEA system. Formate and acetate are transported to the anode as they are negatively charged ions, and neutral molecules, such as ethanol and propanol, are dragged by electro-osmotic force.

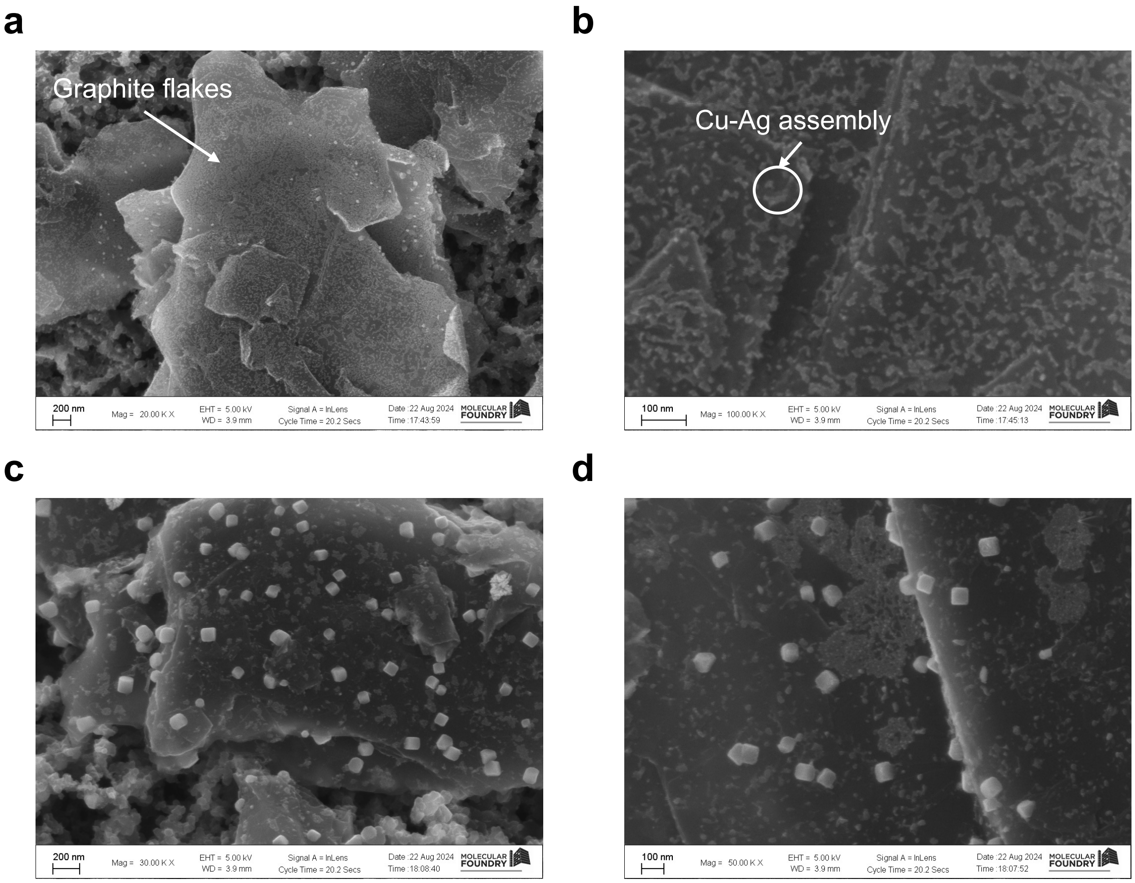

Fig. S2. Morphology of the Cu-Ag tandem electrocatalysts. Scanning electron microscope images of (a-b) as-prepared Cu-Ag tandem electrocatalysts on a carbon paper-based gas diffusion electrode and (c-d) post-electrolysis of Cu-Ag tandem electrocatalysts.

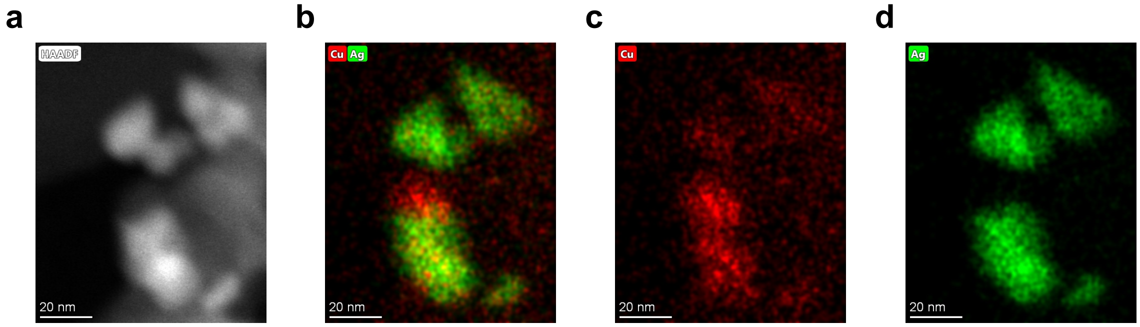

Fig. S3. Morphology of the post-electrolysis of Cu-Ag tandem electrocatalysts. (a) Transmission electron microscope (TEM) image. (b) corresponding Energy-dispersive X-ray spectroscopy (EDX) composition map of Cu (red) and Ag (green). (c) Cu map, and (d) Ag map.

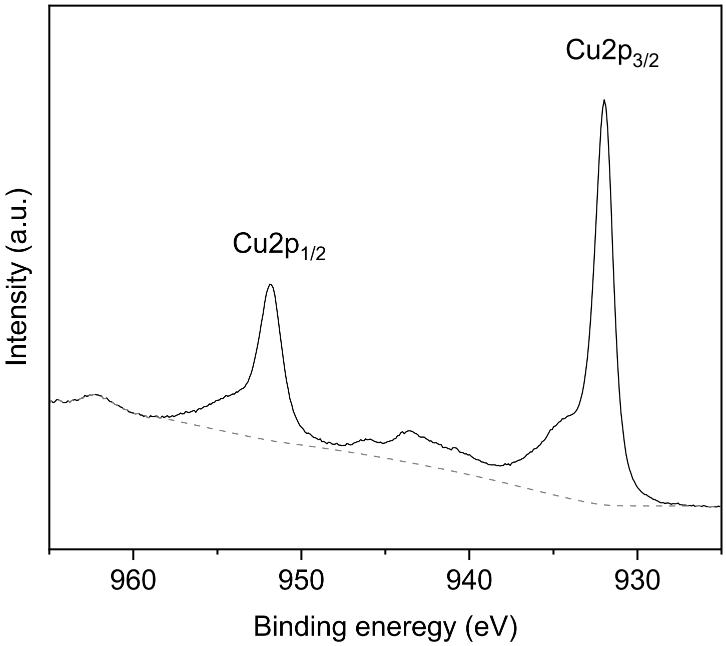

Fig. S4. X-ray photoelectron spectroscopy spectra of pure Cu electrocatalyst.

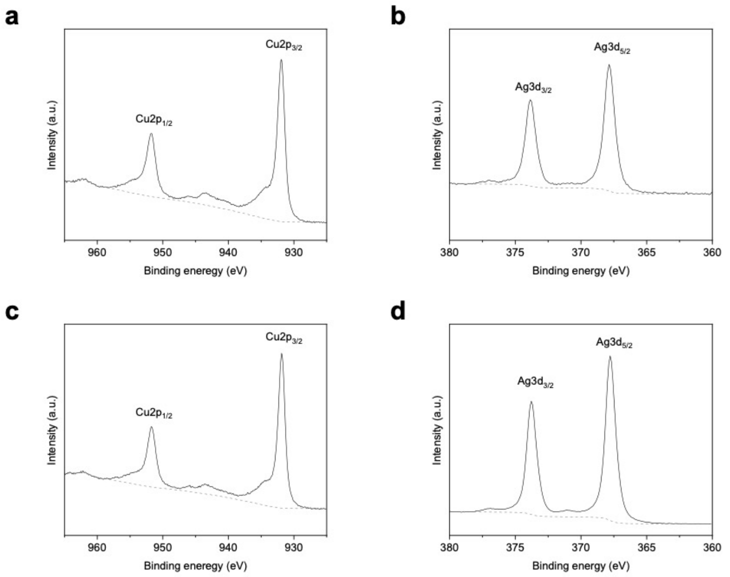

Fig. S5. X-ray photoelectron spectroscopy spectra of (a) Cu of as-prepared Cu-Ag (Ag 20%) tandem electrocatalyst, (b) Ag of as-prepared Cu-Ag (Ag 20%) tandem electrocatalyst, (c) Cu of as-prepared Cu-Ag (Ag 66%) tandem electrocatalyst, and (d) Ag of as-prepared Cu-Ag (Ag 66%) tandem electrocatalyst.

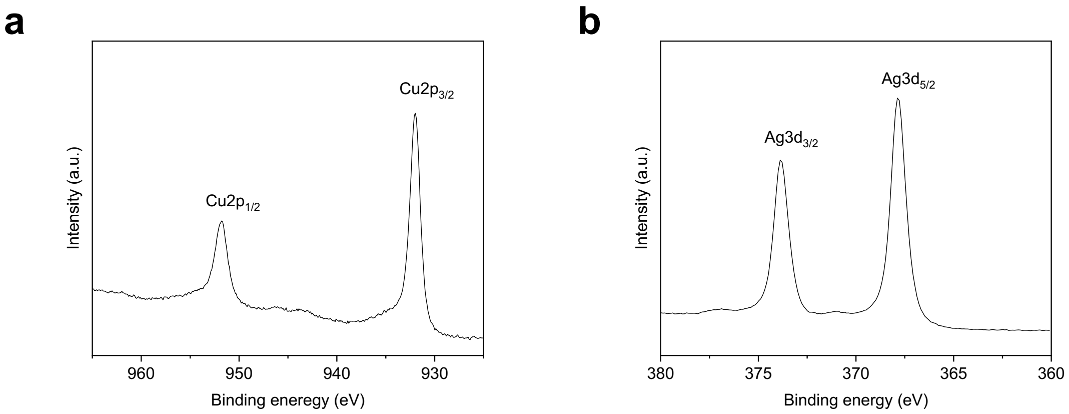

Fig. S6. X-ray photoelectron spectroscopy spectra of post-electrolysis (a) Cu and (b) Ag. No peak shift was observed after electrolysis, indicating that nanoparticle assembly does not originate from electronic interaction between Cu and Ag.

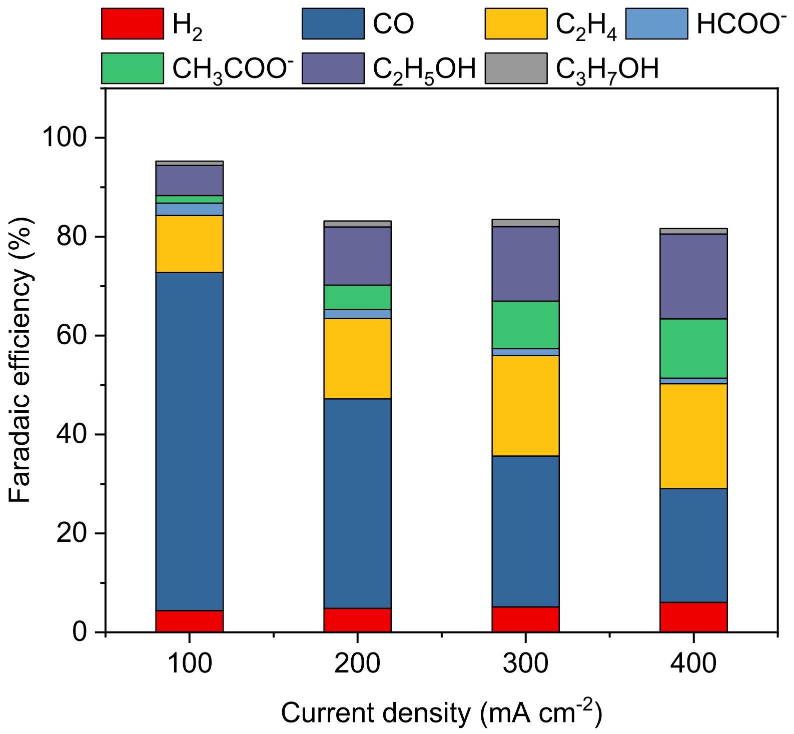

Fig. S7. Product distribution of Cu-Ag (Ag 45%) tandem electrocatalyst in the MEA cell at a CO_2 flow rate of > 3.4 μmol s^{-1} cm^{-2} (5 sccm cm^{-2}).

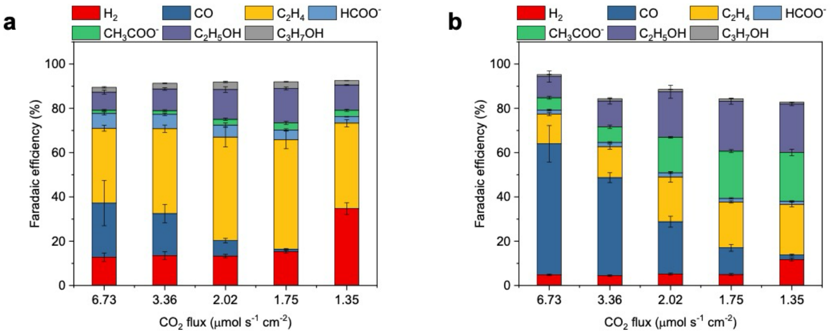

Fig. S8. The product distribution of (a) pure Cu electrocatalyst and (b) Cu-Ag (Ag 45%) tandem electrocatalyst in the range of CO_2 flux from 1.35 μmol s^{-1} cm^{-2} to 6.73 μmol s^{-1} cm^{-2} at applied current density of 300 mA cm^{-2} in the MEA cell.

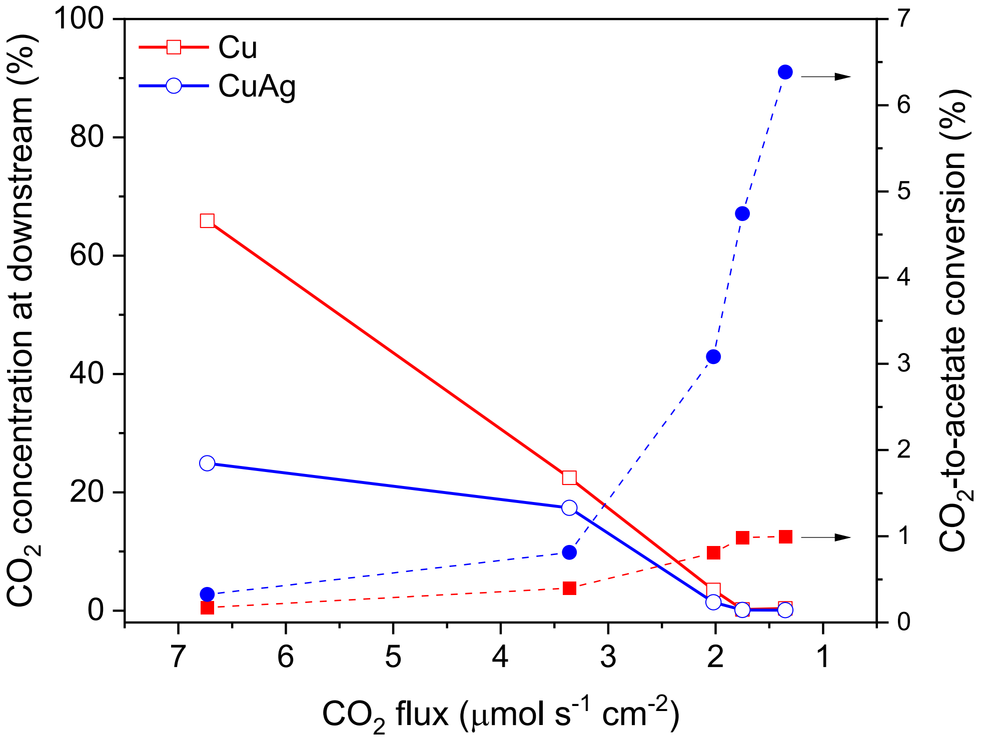

Fig. S9. CO_2 concentration (%) at the cathodic outlet and CO_2-to-acetate conversion ratio (%) for pure Cu electrocatalyst and Cu-Ag (Ag 45%) tandem electrocatalyst in the range of CO_2 flux from 1.35 μmol s^{-1} cm^{-2} to 6.73 μmol s^{-1} cm^{-2} at the current density of 300 mA cm^{-2} in the MEA cell.

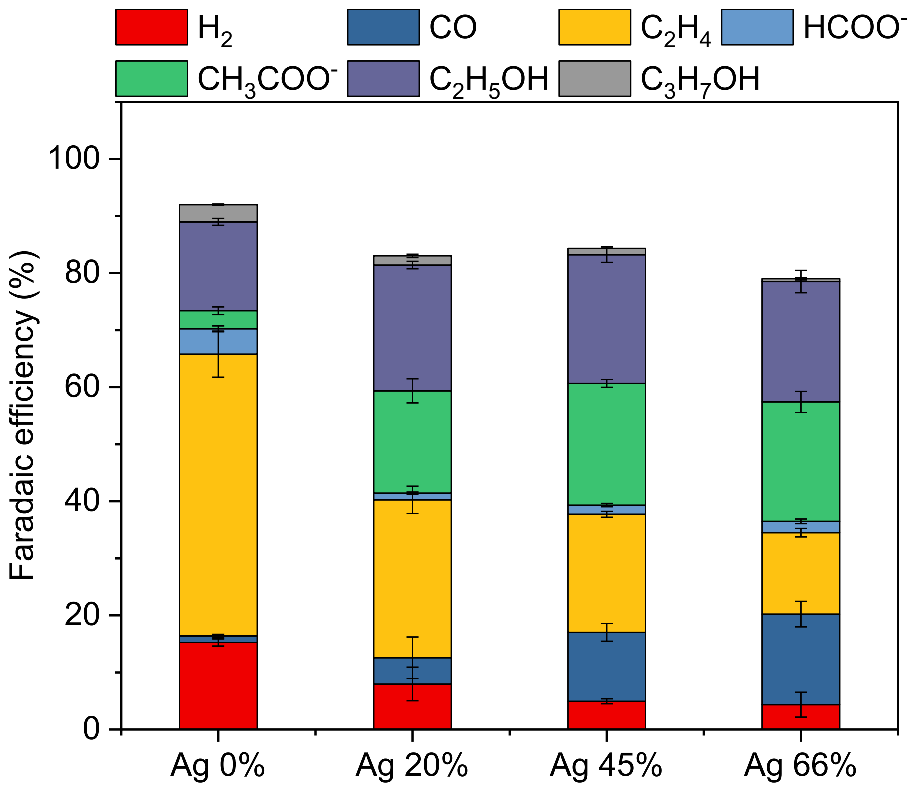

Fig. S10. Product distribution of Cu-Ag tandem electrocatalysts with varying Ag ratio (0%, 20%, 45% and 66%) at applied current density 300 mA cm^{-2} in the MEA cell.

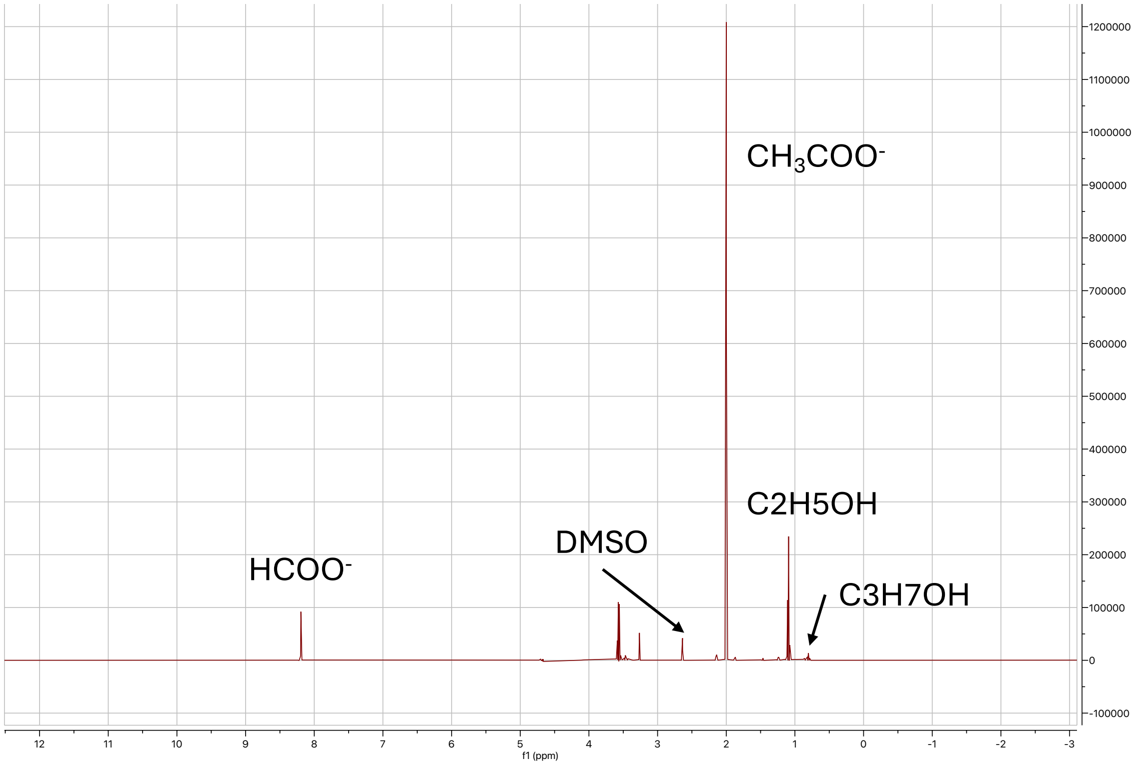

Fig. S11. ^{1}H nuclear magnetic resonance (NMR) spectrum of a mixture of 0.1 M CsH_2PO_4 anolyte and liquid products at an applied current density of 400 mA cm^{-2}. Dimethylsulfoxide (DMSO) is used as a reference chemical.

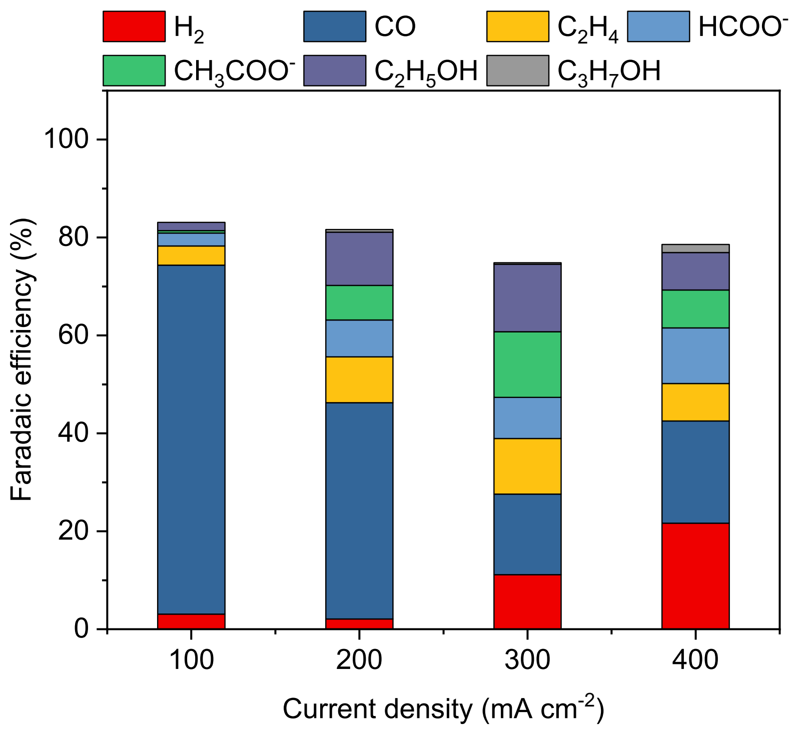

Fig. S12. Product distribution of Cu-Ag (Ag 45%) tandem electrocatalyst with 0.1 M KH_2PO_4 anolyte in the membrane electrode assembly.

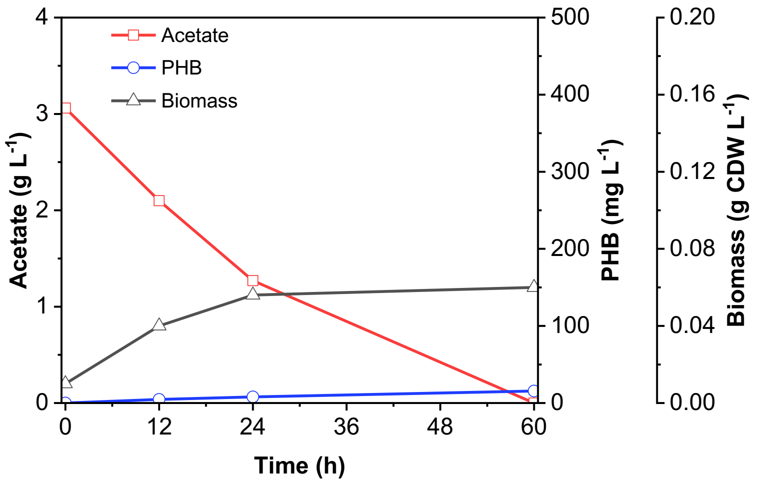

Fig. S13. Time-course bioconversion of electrosynthesized acetate from CsH_2PO_4 to PHB by *Cupriavidus necator*. Acetate consumption (red, left y-axis), PHB production (blue, right y-axis), and biomass accumulation (CDW; black, right y-axis) were monitored throughout the bioconversion process.

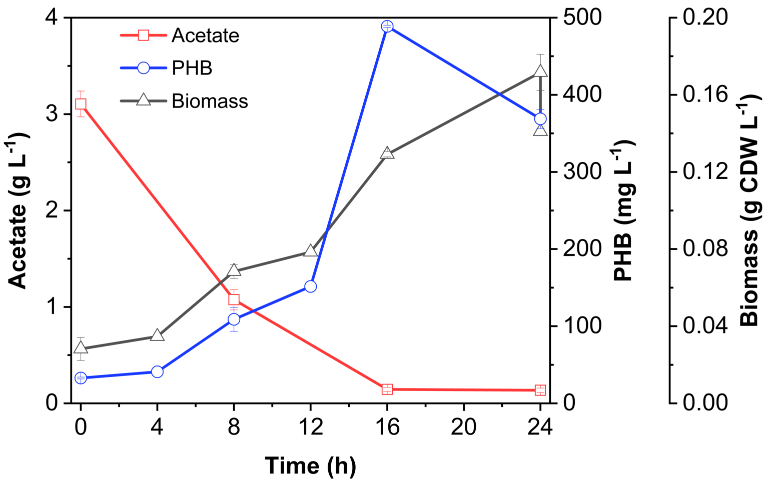

Fig. S14. Bioconversion of standard sodium acetate solution to PHB using *Cupriavidus necator.* Time-course profiles of acetate consumption (red, left y-axis), PHB production (blue, right y-axis), and Biomass (cell dry weight (CDW), black, right y-axis) during the bioconversion process. Data represents mean values ± standard deviations from triplicate experiments. After complete depletion of acetate at 16 hours, the observed decrease in PHB content and simultaneous increase in biomass suggest that the accumulated PHB was degraded and utilized as an intracellular energy source to support cell growth.(8)

	1	2	Lab data
**Electrochemical performance metrics**			
**$V_{full\text{-}cell}$**	3.5 V	3.2 V	3.9 V
**Current density**	400 mA cm^{-2}	400 mA cm^{-2}	400 mA cm^{-2}
**Faradaic efficiency**	30%	56%	26%
**Energy efficiency**	10%	20%	7%
**Electrolysis energy**	153 GJ/tonne of acetate	75 GJ/tonne of acetate	191 GJ/tonne of acetate
**Carbon emission from the bioreactor (kg CO_{2e}/kg polymer)**			
	0.25	0.25	0.25
**Carbon footprint (kg CO_{2e}/kg polymer)**			
**Wind electricity**	1.21	0.72	1.48
**Hydro electricity**	2.31	1.26	2.89
**Solar electricity**	3.66	1.92	4.64

Table S1. The carbon footprint of the CO_2 electrolysis systems with projected metrics and lab data.

	Cu	Ag	Ir
**Concentration**	30 ppb	65 ppb	< 10 ppb

Table S2. The concentrations of inorganic species in a mixture of electrolyte and liquid products. The concentrations were analyzed by inductively coupled plasma optical emission spectroscopy (ICP-OES). A detection limit of ICP-OES is 10 ppb.

	CuAg_1	CuAg_2	CuAg_3
**Cu concentration (mg/L)**	1.48	1.45	0.16
**Ag concentration (mg/L)**	0.63	2.04	0.54
**Cu atomic ratio (%)**	80	55	34
**Ag atomic ratio (%)**	20	45	66

Table S3. The concentrations and atomic ratio of the as-prepared Cu-Ag GDEs. The concentrations were analyzed by ICP-OES.

Cycle	Time (h)	Electrolyte volume (mL)	Acetate concentration (mM)	Ethanol concentration (mM)
**1**	8	24.5	0.219	0.184
**2**	5	19.5	0.167	0.112
**3**	4	20	0.204	0.165
**4**	4	19	0.179	0.133
**5**	6	25	0.199	0.117
**6**	6	20	0.265	0.145
**7**	13	40	0.284	0.151
**8**	5	20	0.162	0.042
**9**	11	40	0.303	0.131
**10**	12	50	0.230	0.085
**11**	5	20	0.198	0.072
**12**	12	50	0.195	0.083
**13**	12	35	0.191	0.051
**14**	12	40	0.185	0.038
**15**	15	45	0.155	0.003
	**129**	**468**		

Table S4. Liquid collection during long-term operations. We changed the electrolyte or catalyst every cycle.

Component	Concentration (per 1L)
DM9 medium	
NH_4Cl	0.075 g
KH_2PO_4	3.0 g
Na_2HPO_4	6.78 g
$MgSO_4 \cdot 7H_2O$	0.49 g
$CaCl_2 \cdot 2H_2O$	0.015 g
Vitamin	1 ml
Trace metal solution SL-6	3 ml
CO_2 electrolyzer downstream	
Electrosynthesized acetate	3 g
KH_2PO_4	2.7 g
Vitamin stock solution	
Biotin	2 mg
Folic acid	2 mg
Pyridoxine × HCl	10 mg
Thiamine × HCl × $2H_2O$	5 mg
Riboflavin	5 mg
Nicotinic acid	5 mg
D-Ca-pantothenate	5 mg
Vitamin B12	0.1 mg
p-aminobenzoic acid	5mg
Lipoic acid	5 mg
Trace metal solution SL-6	
$ZnSO_4 \cdot 7H_2O$	0.1 g
$MnCl_2 \cdot 4H_2O$	0.03 g
H_3BO_3	0.3 g
$CoCl_2 \cdot 6H_2O$	0.2 g
$CuCl_2 \cdot 2H_2O$	0.01 g
$NiCl_2 \cdot 6H_2O$	0.02g
$Na_2MoO_4 \cdot 2H_2O$	0.03 g

Table S5. DM9 medium composition with electrosynthesized acetate.

SI References

1. J. D. Adams, D. S. Clark, Techno-Economic Assessment of Electromicrobial Production of n-Butanol from Air-Captured CO_2. *Environmental Science & Technology* **58**, 7302-7313 (2024).
2. S. L. Dolan, G. A. Heath, Life cycle greenhouse gas emissions of utility-scale wind power: systematic review and harmonization. *Journal of Industrial Ecology* **16**, S136-S154 (2012).
3. O. Edenhofer *et al.*, *Renewable Energy Sources and Climate Change Mitigation: Special Report of the Intergovernmental Panel on Climate Change* (Cambridge University Press, 2011).
4. C. Liu *et al.*, Nanowire–bacteria hybrids for unassisted solar carbon dioxide fixation to value-added chemicals. *Nano letters* **15**, 3634-3639 (2015).
5. M. Gustavsson, S. Y. Lee, Prospects of microbial cell factories developed through systems metabolic engineering. *Microbial Biotechnology* **9**, 610-617 (2016).
6. S. Van Dien, From the first drop to the first truckload: commercialization of microbial processes for renewable chemicals. *Current Opinion in Biotechnology* **24**, 1061-1068 (2013).
7. J. E. Huang *et al.*, CO_2 electrolysis to multicarbon products in strong acid. *Science* **372**, 1074-1078 (2021).
8. L. Zhang *et al.*, A review on enhancing Cupriavidus necator fermentation for poly (3-hydroxybutyrate)(PHB) production from low-cost carbon sources. *Frontiers in bioengineering and biotechnology* **10**, 946085 (2022).
